# Interorganizational Knowledge Transfer in Mass Gatherings: Exploring the Health and Safety Stakeholders’ Perceptions Participating in the Athens Marathon

**DOI:** 10.1017/S1049023X24000219

**Published:** 2024-04

**Authors:** Angeliki Bistaraki, Nikos Stefanopoulos

**Affiliations:** 1. Department of Nursing, School of Health Sciences, Hellenic Mediterranean University, Crete, Greece; 2. Department of Nursing, School of Health Rehabilitation Sciences, University of Patras, Patras, Greece

**Keywords:** Athens, interorganizational knowledge transfer, marathon, mass gatherings, public health

## Abstract

**Introduction:**

Mass gatherings (MGs) usually represent significant challenges for the public health and safety sector of the host cities. Organizing a safe and successful mass event highly depends on the effective collaboration among different public and private organizations. It is necessary to establish successful coordination to ensure that all the key stakeholders understand their respective roles and responsibilities. The inconsistency between the variety of participating agencies because of their different culture can result in delays in decision making. Interorganizational knowledge transfer can improve the success of the event; however, knowledge transfer among professionals and agencies in MGs is not well-documented.

**Objective:**

This study used the 2018 Athens Marathon as the empirical setting to examine how interorganizational knowledge transfer was perceived among the multiple public health and safety professionals during the planning stage of the event.

**Methods:**

Data comprised 18 semi-structured, in-depth interviews with key informants, direct observations of meetings, and documentary analysis. Open coding and thematic analysis were used to analyze the data.

**Results:**

Findings indicated that sharing the acquired knowledge was a necessary and challenging step to create an enabling collaborative environment among interacting organizations. Experiential learning was identified as a significant factor, which helped promote joint understanding and partnership work. Informal interpersonal exchanges and formal knowledge transfer activities facilitated knowledge sharing across organizational boundaries, helping to break down silos.

**Conclusion:**

Interorganizational knowledge transfer is a necessary step to achieve joint understanding and create an environment where interaction among agencies can be more effective. The study findings can be beneficial for organizers of marathons and other mass sporting events to support valuable interorganizational collaboration and conduct a safe event.

## Introduction

Mass gatherings (MGs) are defined as events attended by a sufficient number of people to strain the planning and response resources of the community, state, or nation.^1^ Major areas of public health responsibility involve the provision of health services to spectators and participants, mass-casualty preparedness, disease surveillance and outbreak response, environmental health protection, public information, health promotion, and preparedness for possible chemical, biological, radiological, and nuclear (CBRN) incidents.^2^ During MGs, potential public health risks include communicable diseases, heat- or cold-related illnesses, foodborne and waterborne illness, and mass-casualty incidents.^3^

Marathons are mass sporting events which are prone to various risks, both natural and manmade. The “Athens Marathon, The Authentic” is a tough course of 42,195m, which has turned into the biggest and most important of all long-distance running sports events in Greece over the last years. The organizers prioritize the safety of participants by implementing various measures, including medical support stations along the course, trained personnel to assist runners in case of emergencies, and security personnel to manage crowds and ensure the smooth flow of the event. There are over 30 medical stations along the course staffed with doctors, nurses, and volunteers equipped to handle various medical emergencies. These stations provide services such as hydration, first aid, and medical assessment. Additionally, security personnel are deployed strategically along the course to manage crowds, control traffic, and ensure the safety of both runners and spectators. Integrated emergency response plans are also in place to address various scenarios, including severe weather conditions, accidents, and other emergencies that may arise during the event.

Successful preparation for such an event requires extensive planning and poses unique risk management challenges for event stakeholders, including interagency cooperation and communication, accountability issues, crowd control, medical needs, and weather-related issues.^4,5^ The primary objectives of the public health response system during these events are: (1) to detect and respond rapidly to disease outbreaks, (2) to prevent foodborne and waterborne infectious diseases, (3) to ensure that medical response to individual emergencies and possible mass casualties would be efficient, (4) to respond to CBRN incidents, and (5) to take advantage of MGs as an opportunity to promote health prevention messages.^2,6^

Collaboration between many diverse agencies, even from sectors that do not usually work together, is necessary to ensure that each organization will respond effectively to a potential emergency.^7,8^ One study has highlighted that the cultural differences among emergency services that operate during a mass event and the limited understanding of each other’s norms and procedures are major challenges while planning and implementing such a sporting event.^9^ Literature has shown that it is necessary to establish strong collaboration and coordination systems, supported by interagency agreements, to ensure that all the key stakeholders understand their respective roles.^10–12^ According to Bistaraki and Georgiadis, many participating stakeholders do not understand other agencies’ roles, requirements, and type of language, which may lead to misunderstandings during communication and may increase the level of uncertainty about partners’ responsibilities.^13^

Another study recommended that agencies should conduct exercises (discussion-based or operational-based) to practice plans, policies, and procedures in advance and identify any gaps in knowledge and response capabilities.^14^ Furthermore, the use of a common and unified domain ontology can improve the decision-making process where most of the emergency decisions are dependent on individual experiences and domain knowledge of relevant managerial personnel.^15^ Nonetheless, there is a need for further research in interorganizational knowledge transfer in MGs. Therefore, the purpose of this study was to further explore how interorganizational knowledge transfer was perceived among the multiple public health and safety professionals during the planning stage of the 2018 “Athens Marathon, The Authentic.”

## Methods

### Study Design

A qualitative single, holistic, and exploratory case study design with multiple data sources was used. Case study researchers hold the view that reality is a social construction.^16^ This methodology ensures that the phenomenon under study is not explored through one lens, but rather a variety of lenses, which allows for multiple facets of the issue to be revealed and a holistic understanding of the phenomenon to be reached.^17,18^ The research took place during the planning stage of the 2018 Athens Marathon.

### Setting and Selection of Participants

The study population consisted of organizing stakeholders such as race event staff, law enforcement, emergency managers, Emergency Medical Services, and voluntary organizations. Purposive sampling was employed covering diverse types of key and senior roles.^19^ This method of sampling facilitated detecting the most relevant and knowledgeable participants. The sample size was 18 professionals (Table 1) who belonged to the above organizations. To be eligible for the study, participants had to be willing to participate and have a key role in public health and safety agencies organizing the event. Written informed consent was obtained from all the respondents and their identities remained confidential by using identification numbers. Ethical approval was granted from University of Peloponnese, School of Human Movement and Quality of Life Science Ethics Committee (Sparta, Greece; No 376/23-10-2017).

**Table 1.** Participant Characteristics

Participant No	Gender	Organization
1	M	Event Staff
2	M	Voluntary Organization
3	F	Organizing Committee
4	M	Military
5	M	Organizing Committee
6	M	Police Service
7	M	Police Service
8	M	Ambulance Service
9	F	Ambulance Service
10	M	Ambulance Service
11	M	Emergency Manager
12	M	Medical Staff
13	M	Organizing Committee
14	F	Medical Staff
15	M	Organizing Committee
16	F	Emergency Staff
17	M	Medical Staff
18	M	Emergency Manager

### Data Collection

The study was conducted during the planning stage of the event. Data were collected through semi-structured in-depth interviews, direct observations, and documentary analysis. The most significant advantage gained by using multiple sources of evidence was triangulation where researchers compare different methods and perspectives to help produce more comprehensive findings and delineate the existence of multiple versions of reality.^20^ First, 18 semi-structured in-depth interviews were conducted during the planning stage of the event. This approach provided the opportunity to capture professional experiences of interorganizational knowledge transfer and discuss new topics brought up by the participants. Respondents were encouraged to express their experiences in their own words. The list of the interview questions that was used is provided in Table 2. The interviews occurred in a place mutually agreed by both the lead author and the participant. Interviews were digitally recorded with the participants’ permission and fieldnotes were kept after the interviews, capturing the context and researcher insights and personal thoughts. The average duration of the interviews was 60 minutes.

**Table 2.** Interview Questions

1	Could you provide a brief history of yourself, your position title, years of experience, and your work in the agency?
2	Could you state what was your and the agency’s role and responsibilities regarding the Marathon?
3	Do you think you had a clear job description?
4	Could you describe the skills that were necessary for communicating with other professionals?
5	How did you acquire your knowledge?
6	Are you familiar with other agencies’ culture, roles, and responsibilities?
7	Have you participated in any interagency exercises and what is your feedback?
8	Did all the stakeholders have the same aims?
9	Could you provide an overview of the relationships between staff within and outside of your organization?
10	Could you describe the communication between yourself and the various parties?
11	Were you sharing information informally?
12	Could you describe the process of information sharing that took place among the agencies and give an example?
13	How interagency communication influenced collaboration?
14	Did you use the same terminology across partners? Any encountered problems?
15	What are the main lessons learned?
16	Is there any additional aspect that would be useful for the aim of this study?

Second, direct observations of interagency meetings during the planning stage of the event were conducted. In direct observations, the researcher acts as a complete observer and does not participate in what is being observed.^16,21^ The observations supported the interview data and allowed the examination of interorganizational knowledge transfer as it naturally occurred.^21^ They included observations of seven interagency meetings during the planning phase resulting in 36 hours of field observation. The observations focused on several domains involving describing the physical environment and the participants, examining the process of knowledge transfer including information-sharing interactions, reporting encountered problems and applied solutions, assessing participant opinions outside the formal meeting, and reflecting on the general atmosphere of the process. Finally, a range of documents produced by the agencies such as reports and strategic and procedures manuals were used and analyzed as secondary sources of evidence to complement evidence from other sources.^20^ It was evaluated how these documents contributed to interorganizational knowledge transfer and the extent to which they were truly used by the organizations, or they were simply cosmetic manuals.

### Data Analysis

Due to the comprehensive nature of the data collected in this study, extensive time was required for thorough analysis and interpretation. Additionally, the unforeseen challenges posed by the COVID-19 pandemic further contributed to the delay in finalizing the results. All interviews were transcribed verbatim, and then the transcribed interview word files were imported into NVivo 12 qualitative data analysis Software (QSR International Pty Ltd; Doncaster, Australia). Transcriptions of interviews, observation fieldnotes, and documents were coded using thematic analysis.^22^ Analysis was mainly open-ended by which issues were identified as they emerged. After completing the coding phase, the research team discussed each code separately to better understand the meaning. The final key components of interorganizational knowledge transfer were decided through discussion with the whole research team. Trustworthiness was assured through the methods of audit trail, triangulation, member check, and peer review of data analysis.^21^

To maintain an audit trail, detailed fieldnotes were taken during each interview capturing participant responses, non-verbal signs, and relevant contextual information. These notes were recorded in a structured format, including date, time, location, and the researcher’s reflections on emerging themes. To ensure the credibility of these findings, data source triangulation was employed encompassing multiple methods of data collection. Semi-structured interviews provided rich insights into participant experiences, while direct observation facilitated an in-depth understanding of the phenomenon within its natural context. Additionally, documentary analysis of relevant records complemented the primary data sources, offering valuable contextual information. Transcripts were returned to participants for verification. Participants were also presented with the final description of the themes and asked to provide feedback on the accuracy and interpretation of the results. None of them provided negative feedback to the principal investigator. Finally, to ensure the trustworthiness of the findings, external peer review was sought from independent experts in the field. Two reviewers were selected based on their expertise in qualitative research methodology and familiarity with the subject addressed in the study. Each reviewer received an anonymized copy of the research manuscript and was asked to provide feedback on various aspects of the study, including the rigor of data collection and analysis procedures and the interpretation and presentation of findings.

## Results

In the process of data analysis, the two main themes that emerged were: *The Challenge of Intra- and Interorganizational Knowledge Transfer;* and *Mechanisms Facilitating Knowledge Transfer,* which the latter included three sub-themes: (1) experiential learning, (2) codified knowledge, and (3) face-to-face interaction. These themes represented those areas participants identified as crucial to influencing interorganizational knowledge transfer and they are discussed below in detail accompanied by exemplar data quotations.

### The Challenge of Intra- and Interorganizational Knowledge Transfer

An important consideration of the professionals who participated in the study was to ensure that they would be aware of the roles and working practices of other agencies during an emergency, and how the actions of different services would be integrated to collaborate effectively. The knowledge that was acquired by individuals using a variety of methods needed to be shared with other individuals or groups of people within and across agencies in order to be applicable and useful during their collaboration rather than remain just personal knowledge. A quote from a police officer highlighted this concern:

There are other people into the party and so you need to make sure that they understand […], you can’t just rely on the individual cause, as I’ve said, the individual can go (Participant 6).

Organizations had to rely on knowledge acquired by their personnel to be able to develop the capabilities needed for such an event. Even though many agencies had sent their staff to other marathons in order to gain both tacit and explicit knowledge, in some cases, their learning was not shared. Their experience and constructive feedback would be useful for the professionals and organizations participating in the event to reflect on the collaborative skills and processes that actors used in other marathons. Some respondents suggested that it was a great challenge to integrate the individual learning into shared learning. One participant from the event staff noted during the interview:

I think we should be more mindful from lessons from other events and the fact that we haven’t, or maybe some colleagues have visited London marathon, they visited other countries but haven’t shared the learning; that’s a failure in my view, is that we haven’t shared this experience (Participant 1).

Similarly, one respondent from a voluntary organization mentioned:

We sent a team to London, we send about three people to go and see what the voluntary organizations are doing there, but we have no idea what happened because they haven’t told us anything from there (Participant 2).

Unfamiliarity with other agencies’ practices and structures was regarded as a crucial element in acquiring information and reaching a joint understanding among the agencies. Organizations that did not know each other well lacked an understanding of the other’s roles and objectives, which made knowledge transfer more difficult. Moreover, some respondents mentioned that some organizations could not absorb the information received from agencies they did not know well, which resulted in the misinterpretation of roles and conflicts during their collaboration. Therefore, unfamiliarity among organizations may have hindered the transfer of their tacit understandings, which was critical to the development of their relationship. One respondent from the Organizing Committee shared her perspective concerning the level of understanding between agencies:

So, they don’t know how we would normally work. So, that’s an adding complication; it’s, it’s probably one of the most challenging things because you are working with people who don’t understand your business as usual (Participant 3).

### Mechanisms Facilitating Knowledge Transfer

*Experiential Learning—*Experiential learning, in which participants learned through experience, helped professionals to understand the other agencies’ roles and practices. It was widely reported by the respondents in this study that conducting interagency meetings and exercises during the planning stage of the event was a useful, interactive way of accessing new knowledge by other partners. One advantage was that professionals from different organizations had increased opportunities to meet individuals from other agencies and explore their knowledge. According to many respondents, having the opportunity to meet people from other organizations, understand their views, and build relationships enabled them to share their experiences and expand their tacit knowledge. Creating new contacts from other organizations and building a strong information exchange network helped participants to learn the other agencies’ roles and how they would work together. The underlying mechanism through which learning was enabled was that professionals created both formal and informal relations, which increased the number of interactions across organizational boundaries and knowledge was transferred more frequently. As three respondents reported:

Because we are involved in the emergency planning…, in meetings and exercises, we get new, in every exercise generally a new of information will come, a new contact will be made. And that’s something we can use then to build on… (Participant 11).

There is always good to come out of the exercises, even if it’s making a new friend, making new contact, understanding somebody’s role (Participant 4).

I think… the biggest benefit we get is we get to sit with other agencies that we work with and get to understand how they operate (Participant 7).

During these exercises, professionals interacted with each other by being in a physical contact and had constant dialogues about how they would operate during the event. In this way, individuals were able to absorb other viewpoints and learned to “speak” the other’s language. Having all the individuals gathered in one place with the specific goal of learning from each other contributed positively to interagency knowledge transfer. More specifically, the physical co-location helped professionals to establish a clearer sense of the connectivity and interactivity that would take place among them during the event and create a more collaborative environment. The following quotes described interviewees’ perceptions on this issue:

So, through these exercises…, through consultation, we’re looking at refining those roles and making those better and… we’ll take advantage of to make it easier to define what that role is, to make it better (Participant 8).

I think again, that through the testing and exercising, we have quite clear roles and responsibilities so all of us know what we do; I think it’s quite clear (Participant 12).

Furthermore, many participants highlighted the importance of understanding their own organization’s environment and structure before learning the practices of other agencies. Internal (intraorganizational) exercises had the ability to examine whether individuals had transferred the knowledge their agency had provided through formal training and workshops to tacit knowledge which was used within professional work practices. The absence of internal knowledge could create some difficulties in knowledge transfer to other partners. Most of the respondents mentioned that in order to collaborate with other agencies, professionals needed to know their internal way of working, and internal exercises were considered to be a great enabler in this process. Moreover, new employees had the opportunity to learn key organizational knowledge, which allowed them to understand how their agency would operate during the event. Therefore, internal exercising appeared to be necessary to ensure that individuals had the relevant knowledge and capabilities to achieve timely decision making with different agencies during a public health or safety incident at the event. The following quote illustrated the importance of exercises in internalizing the explicit knowledge provided:

We did exercises afterwards to make sure the training had worked… testing all of the command-and-control procedures for the Marathon… testing the plans, preparedness… absolutely necessary (Participant 9).

*Codified Knowledge—*Codified knowledge was deemed as a useful mechanism by which respondents acquired explicit knowledge including the structures and procedures of other services. This method allowed participants to understand external (to their organization) knowledge and assisted them to adapt to the interagency environment of the event. Several agencies produced short training packages to familiarize professionals within and across organizations with key knowledge including the roles and responsibilities of personnel and technical information and information sharing procedures that would be followed. Consequently, professionals would develop a shared understanding of each agency’s position and would feel part of an integrated collaborative network. One Emergency Manager noted during the interview:

Formal education is quite good to give you that background of understanding and the skills and the judgment that you need for your role when you come to the meetings and the relationships and that activities with other colleagues (Participant 18).

It was also evident throughout the data that joint training facilitated the externalization of knowledge among the agencies during the planning stage. During such training, the combination of experts who shared their tacit knowledge, along with the provision of documentation which included more explicit information, facilitated the development of integrated plans by the agencies. During joint training, the experiences and technical knowledge of experts were transferred with ease to all the organizations, which strengthened their integrated approach to the event. The following quote gave an example of the benefits of joint training:

So, there will be four training dates in the next six weeks, and we have invited every organization to take this training course and we are providing a training pack with follow up expertise to help to develop these plans (Participant 5).

Adding to the previous codified knowledge, each organization acquired static stocks of knowledge derived from its institutional processes including the rules, norms, procedures, and structures that have been followed for all the years of their existence. According to many respondents, exploiting the existing knowledge of their organization enabled their response to their partners’ needs. Comprehending this basic knowledge also allowed them to recognize the assumptions that shaped the operations of their organization and therefore be in a better position to apply it to the interagency environment of the event. Furthermore, using the same procedures instead of developing new ones increased professional confidence in their activities and, in turn, supported better interorganizational knowledge transfer. Two participants noted:

We have to be based on what knowledge we got (Participant 10).

A lot of it is coming through our knowledge… using existing knowledge and existing training and just make it specific to the new demands that Marathon will bring (Participant 14).

*Face-to-Face Interaction—*Face-to-face communication was another mechanism that organizations used in order to acquire and share existing knowledge. Sometimes individuals had difficulties in understanding the other agencies’ objectives and priorities. Many interviewees indicated that organizations acknowledged this gap and focused their learning efforts on explicit information transferred from other actors. For example, sharing a single location with other agencies and face-to-face interaction during an exercise, or sharing information about an incident that was managed by more than one agency during the planning phase, led to a shared learning of the processes used by each agency. When organizations exchanged information regarding their aims and practices, agency boundaries were clarified. As one respondent reported:

We don’t always understand what an organization is, what an organization may do… but as long as is communicated to us what that priorities are, then that helps us to say, well ok, you know, this is what they’re doing and therefore, you know, it helps us to work, to respond to that and make sure that we don’t tell them what they are doing (Participant 16).

Specifically, many participants argued that regular informal communication between them in the planning phase helped them to build stronger relationships and exchange useful information around the tacit components of their knowledge. It was thought to be a useful method to access the partner’s experience and specialized knowledge. Frequent informal interaction allowed individuals to exchange complementary knowledge of the public health and safety domain and minimize both parties’ assumptions regarding their roles. In this way, they learned how to collaborate, even though they had different backgrounds and experiences:

But that’s where we all learned each other’s abilities, capabilities, capacities, that we used to do a huge amount of networking as well as normal day to day business (Participant 17).

I’ve met with the medical manager, and I had some kind of conversations with him… and I think that was the better way to convey some of the informal learning (Participant 13).

## Discussion

During the events of the 2018 Athens Marathon, nearly 55,000 runners took part with around 150,000 companions as well as approximately 40,000 spectators – numbers which constitute a big challenge for organizers and involved organizations. Achieving a successful mass event highly depends on the effective provision of public health and emergency services that are often provided by different agencies.^9–13^ Thus, poor collaboration and knowledge sharing between these organizations can result in delays in decision making.^23–28^ This study explored how interorganizational knowledge transfer was perceived among the multiple health and safety professionals during the planning stage of the 2018 Athens Marathon. Consistent with the literature, unfamiliarity with the other agencies’ practices is a crucial element in acquiring information and reaching a joint understanding among them.^9,29^ However, as highlighted in this study, even though organizations had to rely on knowledge acquired by their personnel to develop the capabilities needed for such an event, it was a great challenge to integrate the individual learning into shared learning within the organization.

Based on the data analysis, this study emphasized that experiential learning that takes place in interagency meetings and exercises during the planning stage of the event and creating new contacts was a useful, interactive way of accessing new knowledge by other partners.^13,14,29–31^ In line with existing literature, the physical co-location during meetings and exercises helped professionals to establish a clearer sense of the connectivity and contributed positively to interagency knowledge transfer.^9,29,32^ A crucial aspect emphasized by the findings of this study is that the absence of intraorganizational knowledge could create some difficulties in knowledge transfer to other partners. Internal exercising appeared to be necessary to ensure that old and new employees understand key organizational knowledge and comprehend how their agency would operate during the event. Participants also suggested that frequent informal interaction helped them to build stronger relationships and exchange useful information around the tacit components of their knowledge.

The development of short training packages produced by the key stakeholders was considered to be essential to familiarize professionals within and across organizations with key knowledge, including the roles, responsibilities, and information sharing procedures. As described in previous studies, joint training facilitated the externalization of knowledge among the agencies during the planning stage and reduced inconsistencies in terminology.^14,15^ The current study highlights that exploiting the existing knowledge of each organization, including the rules, norms, procedures, and structures, and using the same procedures instead of developing new ones increased professional confidence in their activities and, in turn, supported better interorganizational knowledge transfer.

## Limitations

The study used established approaches to enhance the validity of the findings.^33–35^ However, this study has limitations which need to be acknowledged. One limitation involved the research setting of the study since the authors explored only the perspectives and experiences of the 2018 Athens Marathon stakeholders. Marathons represent typical MGs, but other types of smaller or bigger mass events also exist, such as the Olympics and religious festivals. Consequently, to further examine the issue of interorganizational knowledge transfer in a MG, research in other settings is recommended. Studying the unique setting of the Athens Marathon limits the transferability of the findings, and therefore, the data should be transmitted with great caution to other contexts. Another potential limitation is that data were collected only during the planning stage of the event. Collecting data also during the implementation stage and after the completion of the event would allow further insights and contribute to a broader understanding of the phenomenon under study. However, because the sample in this study included representatives from all the key stakeholders involved in the marathon, it was deemed to be adequate for the current research problem.

## Conclusion

The Athens Marathon attracts runners from around the world, making it a truly global event. Participants come to experience the historic course and be part of one of the most iconic races in the running community. This marathon event has consistently gathered positive feedback from participants, spectators, and sports enthusiasts world-wide. This study explored how interorganizational knowledge transfer in a MG such as the marathon was perceived among the multiple health and safety professionals during the planning stage of the event. As these events bring together thousands of participants and different organizing agencies, interorganizational knowledge transfer is a necessary step to achieve joint understanding and to create an environment where interaction among agencies can be more effective. The findings suggest that interorganizational knowledge transfer was a challenge during the 2018 Athens Marathon and recommend three mechanisms facilitating this procedure: (1) experiential learning, (2) codified knowledge, and (3) face-to-face interaction. Knowledge transfer can improve the coordination and collaboration between the several stakeholders. This study’s findings may assist future event planners of marathons, or other similar events, to facilitate better knowledge management and decision-making procedures.
